# Study on the Preparation of Diamond Film Substrates on AlN Ceramic and Their Performance in LED Packaging

**DOI:** 10.3390/mi16091029

**Published:** 2025-09-08

**Authors:** Shasha Wei, Yusheng Sui, Yunlong Shi, Junrong Chen, Tianlei Dong, Rongchuan Lin, Zheqiao Lin

**Affiliations:** 1 College of Marine Equipment and Mechanical Engineering, Jimei University, Xiamen 361021, China; 200461000025@jmu.edu.cn (S.W.); 15264223550@163.com (Y.S.); syl15064717701@163.com (Y.S.); dtl1377853727@163.com (T.D.); 2 Xiamen Nenggu Coating Technology Co., Ltd., Xiamen 361021, China; junrongchen75@gmail.com

**Keywords:** AlN ceramics, diamond film, substrate heat dissipation

## Abstract

Aluminum nitride (AlN) ceramic materials have relatively low thermal conductivity and poor heat dissipation performance, and are increasingly unsuitable for high-power LED packaging. In this study, diamond films were deposited on AlN ceramic substrates by microwave plasma chemical vapor deposition (MPCVD). The effects of different process parameters on the crystal quality, surface morphology and crystal orientation of diamond films were studied, and the high thermal conductivity of diamond was used to enhance the heat dissipation ability of AlN ceramic substrates. Finally, the junction temperature and thermal resistance of LED devices packaged on AlN ceramic–diamond composite substrate, AlN ceramic substrate and aluminum substrate were tested. The experimental results show that compared with the traditional aluminum and AlN ceramic substrates, AlN ceramic–diamond composite substrates show excellent heat dissipation performance, especially under high-power conditions.

## 1. Introduction

Light-emitting diodes (LEDs), as a representative of next-generation solid-state light sources [1,2], offer numerous advantages such as high luminous power, long lifespan, energy efficiency, and environmental friendliness. They have been widely adopted in various applications, including indoor and outdoor lighting, display backlighting, and automotive headlights. However, as LEDs evolve toward higher density and greater power, the heat generated by the chips has increased significantly. Studies indicate that only 20–30% of the input power is converted into light, while the remaining 70–80% is dissipated as heat [3,4]. If this heat is not effectively removed during operation, it accumulates within the chip, leading to a rise in the LED junction temperature. This, in turn, severely impacts the LED’s lifespan, luminous efficiency, and light output [5,6]. As a critical component in the thermal management pathway of high-power LEDs, the performance of the heat dissipation substrate plays a pivotal role in determining the overall effectiveness of the LED.

Aluminum nitride (AlN) ceramics [7,8,9,10], known for their excellent overall performance and cost-effectiveness, are among the most commonly used materials for packaging substrates [11,12]. The thermal conductivity of AlN ceramics is between 180–230 W/m·K, which is much higher than that of traditional ceramic materials such as alumina (Al_2_O_3_), but there is still a big gap compared with diamond (>2000 W/m·K). In addition, the thermal conductivity of AlN ceramics will decrease significantly with the increase in temperature, which limits its application in high-power electronic devices to some extent. A promising approach to enhance the performance of AlN ceramics is surface coating with suitable materials. Diamond, with its exceptionally high thermal conductivity, low thermal expansion coefficient, excellent chemical stability, and outstanding mechanical properties, is an ideal coating material [13,14]. Depositing diamond films on AlN ceramic surfaces using chemical vapor deposition (CVD) [15,16] is a cost-effective and practical solution.

Various methods are available for preparing CVD diamond films, including hot filament chemical vapor deposition (HFCVD), microwave plasma chemical vapor deposition (MPCVD), direct current plasma chemical vapor deposition, combustion flame CVD, and direct current arc plasma jet CVD [17,18]. Among these, MPCVD stands out as the preferred method for producing high-quality diamond films [19,20]. This is due to its use of electrodeless discharge, which generates a pure and uniform plasma through electromagnetic wave excitation [21,22]. Additionally, MPCVD offers smooth and continuous control over power and pressure, enabling precise temperature regulation [23,24]. The properties of diamond films [25,26] are closely tied to their preparation process [27,28,29], making the optimization of growth parameters [30] essential for achieving high-quality films.

In this study, high-quality diamond films were grown using MPCVD, and the influence of process parameters on diamond film growth was investigated to identify the optimal fabrication conditions. The diamond films were then metallized using photolithography and magnetron sputtering techniques [31,32,33] and applied in the packaging of high-power LED devices [34]. Comparative experiments were conducted to evaluate the thermal performance of AlN ceramic substrate diamond film substrates against traditional aluminum and AlN ceramic substrates. The junction temperature of LED devices packaged with these substrates was measured under varying currents, and the thermal resistance of each substrate was analyzed. This comprehensive assessment highlights the superior heat dissipation capabilities of AlN ceramic substrate diamond film substrates.

## 2. Experimental

### 2.1. Preparation of Diamond Films

The Ø20 mm × 0.8 mm AlN ceramic substrate was selected in the experiment, the AlN ceramic substrates [2,3,4] were ultrasonically cleaned in a hydrofluoric acid solution for 5 min to remove surface oxides. Subsequently, the substrates were sequentially cleaned with acetone, anhydrous ethanol, and deionized water, each for 5 min, to eliminate organic impurities. The substrates were then immersed in a diamond powder suspension (particle size W0.25) with the growth surface facing upward and ultrasonically treated for 30 min. Finally, the substrates were rinsed with deionized water to remove excess diamond powder and dried with nitrogen gas for further use.

Diamond films were deposited on the AlN ceramic substrates using microwave plasma chemical vapor deposition (MPCVD) [9]. The microwave source operated at a frequency of 2.45 GHz, and the growth gases included 99.999% hydrogen (H_2_), 99.99% methane (CH_4_), and 99.999% oxygen (O_2_). Under the deposition conditions listed in Table 1 (with methane flow accounting for 4% of the total gas flow), diamond films with a thickness of 531 μm were successfully deposited on the AlN ceramic substrates after 150 h of growth.

### 2.2. Fabrication of AlN Ceramic Substrate Diamond Thin-Film Base and LED Packaging Testing

**Figure 1:**
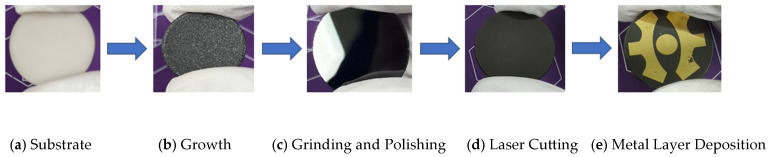
Fabrication Process of AlN Ceramic Substrate Diamond Thin-Film Base.

**Figure 2:**
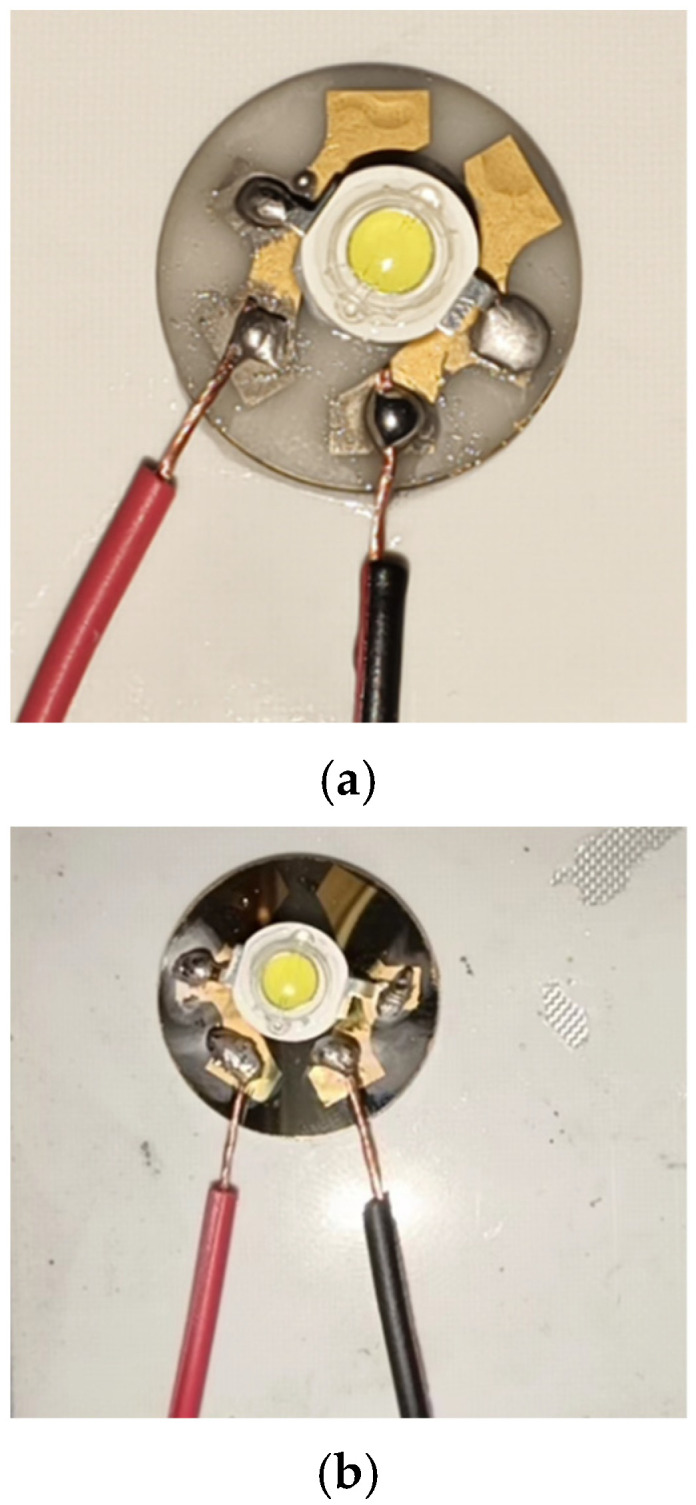

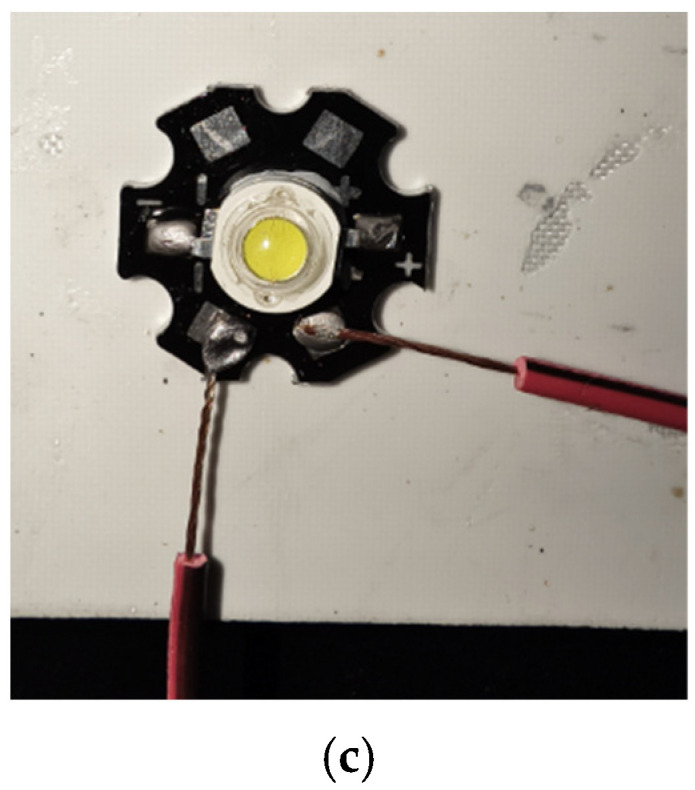
LEDs Packaged with Three Different Substrates. (**a**) AlN Ceramic Substrate; (**b**) AlN Ceramic Substrate Diamond Thin-Film Base; (**c**) Conventional Aluminum Substrate.

After fabricating the diamond thin film on the AlN ceramic substrate using the MPCVD method, a metallization (Magnetron sputtering) process is required to form a conductive metal layer that electrically connects with the LED device, as shown in Figure 1. The fabrication process of the AlN ceramic substrate diamond thin-film base and LED packaging [30] mainly includes grinding and polishing, laser cutting, photolithography, magnetron sputtering, and welding, The final thickness is about 10 mm. To comprehensively evaluate the performance of the AlN ceramic substrate diamond thin-film base [20], conventional aluminum substrates and AlN ceramic substrates (undergoing the same metallization process as the AlN ceramic substrate diamond thin-film base) with similar dimensions were selected as control groups. As illustrated in Figure 2, LED devices (3 W cool white LED lamp beads) were packaged using the same welding process. The study analyzes the junction temperature and thermal resistance of LED devices with the three different substrate types under varying current conditions.

### 2.3. Performance Characterization

The surface morphology of the diamond thin film was characterized using a Sigma500 high-resolution field-emission scanning electron microscope (FE-SEM) manufactured by Zeiss, Germany. The crystal orientation and other structural information of the diamond thin film were analyzed using a SmartLab series X-ray diffractometer (XRD) produced by Rigaku, Japan. The crystal quality of the diamond thin film [24] was evaluated with an Alpha300 confocal Raman spectrometer manufactured by WITec, Germany, utilizing a laser wavelength of 532.287 nm.

The junction temperature of LED devices is measured with the Ulid Uti120S infrared imager made in Dongguan, China. The temperature measurement range is −20~400 °C, and the accuracy is 2 °C.

## 3. Results

### 3.1. Effect of Methane Concentration on Diamond Thin-Film Growth

During the MPCVD process for diamond thin-film fabrication, the methane concentration (the ratio of methane to hydrogen flow) [21,22] directly influences the proportion of atomic hydrogen and various carbon-containing active species in the plasma. The concentration level determines both the quality of the diamond thin film and the deposition rate.

Goodwin et al. [35] found that the quality and deposition rate of the diamond thin film can be expressed by the following equation:

(1) $$\begin{array}{c} G=1.5\times 10^{11}\frac{\left[{CH}_{3}\right]\left[H\right]}{3\times 10^{-9}+\left[H\right]} \end{array}$$

(2) $$\begin{array}{c} \left[def\right]=\frac{G}{{\left[H\right]}^{2}} \end{array}$$

where:

*G* represents the deposition rate of the diamond thin film.

*[CH_3_]* and *[H]* represent the concentrations of carbon-containing active species (CH_3_ is generally considered an effective precursor for promoting diamond growth) and atomic hydrogen, respectively.

*[def]* denotes the concentration of defects in the diamond thin film.

Under normal circumstances, an increase in methane concentration significantly enhances the concentration of effective carbon-containing active species. As seen from Equations (1) and (2), the concentration of these active species is linearly proportional to the deposition rate of the diamond thin film. However, in terms of film quality, a higher concentration of atomic hydrogen can effectively reduce the defect density in the film. Therefore, it is crucial to determine an optimal methane concentration range that maximizes the deposition rate while ensuring high-quality diamond thin films. In this section, without altering other process parameters, we investigate the effects of different methane concentrations (2%, 3%, 4%, and 5%) on the growth quality, surface morphology, and deposition rate of diamond thin films. The specific experimental parameters are listed in Table 2.

**Figure 3:**
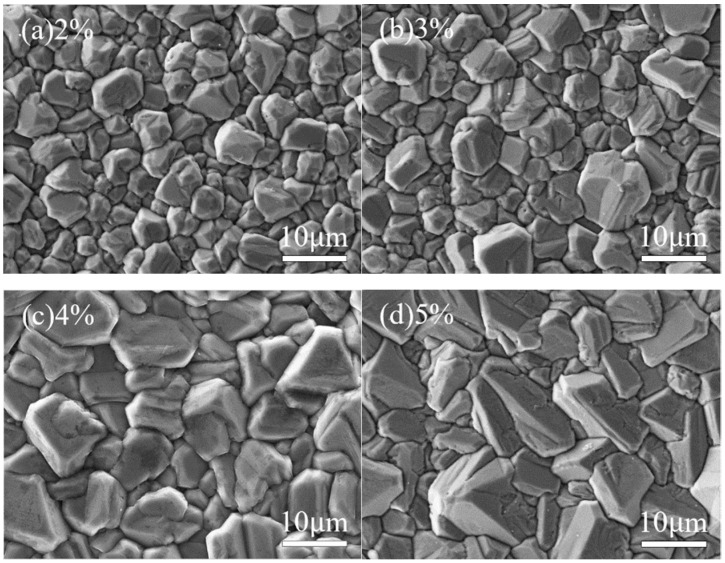
SEM Images of Diamond Thin Film Surface Prepared at Different Methane Concentrations.

**Figure 4:**
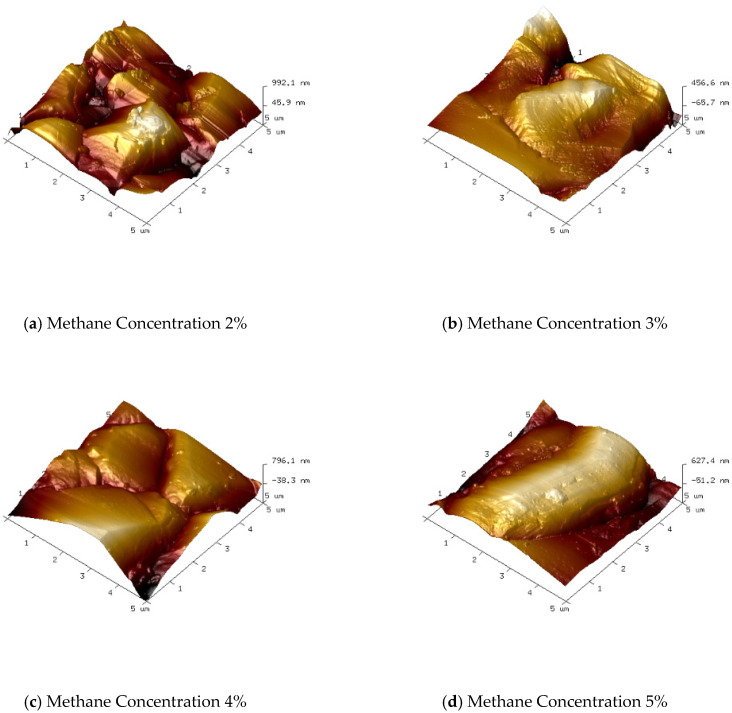
3D AFM Images of Diamond Thin Film Surface Prepared at Different Methane Concentrations (5 µm × 5 µm).

At different methane concentrations, the surface morphology of the diamond thin film exhibits significant differences. Figure 3 and Figure 4 show the surface morphology SEM images and corresponding 3D AFM images of diamond thin films prepared under various methane concentrations. As observed, with the increase in methane concentration, the grain size of the diamond thin films gradually increases.

As shown in Figure 3a and Figure 4a, when the methane concentration is 2%, the diamond thin film is continuous and dense, with a surface roughness of Ra = 136 nm. However, the grain size is small (with an average size of about 5 µm), and the orientation is disordered with no obvious facets. This is due to the low methane concentration, which results in a relatively low number of carbon-containing active species in the plasma. Consequently, the driving force for diamond thin-film growth is weak. Moreover, the excess atomic hydrogen in the plasma, besides etching non-diamond phases like graphite, also etches the diamond thin film, which inhibits its growth.

As shown in Figure 3b and Figure 4b, when the methane concentration is increased to 3%, the number of carbon-containing active species in the plasma increases, and the diamond thin film grain profiles begin to emerge. The surface roughness increases, with Ra = 151 nm. However, due to the still relatively low methane concentration, the grain size increases (average size around 7 µm), but the uniformity remains poor. When the methane concentration is raised to 4%, as seen in Figure 3c and Figure 4c, the grain size of the diamond thin film becomes more uniform (average size around 10 µm), with clear profiles and distinct grain boundaries. The increase in crystal size causes the surface roughness to increase, with Ra = 163 nm. When the methane concentration reaches 5%, the diamond thin film grains take on an elongated shape, hindering the growth of other grains, resulting in poor grain size uniformity and even secondary nucleation phenomena. The primary reason for this is the increased methane concentration, which leads to a higher concentration of carbon-containing active species in the plasma. The excessive carbon saturation causes the diffusion rate of these active species on the film surface to be lower than the rate of secondary nucleation.

Using a high-precision thickness gauge, the growth rates of diamond thin films under different methane concentrations were measured. The growth rates corresponding to methane concentrations of 2%, 3%, 4%, and 5% were 2.3 µm/h, 3.1 µm/h, 4.1 µm/h, and 4.9 µm/h, respectively. It can be observed that the growth rate of the diamond thin film is linearly related to the methane concentration. This is primarily because the growth process of the diamond thin film is a dynamic process involving the deposition of the diamond phase and the etching of non-diamond phases like graphite. When the chamber pressure and deposition temperature are constant, the number of carbon-containing active species reaching the growth surface of the diamond thin film depends solely on the methane concentration. The higher the methane concentration, the greater the number of carbon-containing active species, resulting in a significant increase in the growth rate of the diamond thin film as the methane concentration increases.

**Figure 5:**
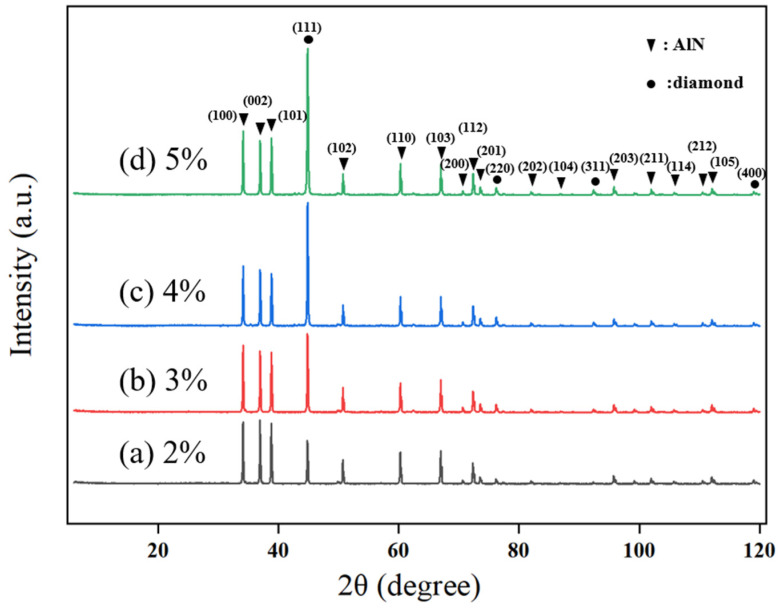
XRD Patterns of Diamond Thin Films Prepared at Different Methane Concentrations.

Changes in methane concentration not only affect the surface morphology and crystal quality of the diamond thin film but also play a role in controlling the crystal orientation. To obtain a more intuitive understanding of the crystal orientation of the diamond thin film, XRD characterization was conducted for diamond thin films prepared under different methane concentrations. To avoid the influence of film thickness on the measurements, each set of control experiments used the thinnest sample for XRD plane adjustment, and the test results are shown in Figure 5.

It can be observed that four crystal orientations are derived during the growth of the diamond thin film: (111), (220), (311), and (400), with (111) being the dominant growth orientation. At a methane concentration of 2%, the diamond diffraction peaks are relatively weak, indicating that the diamond thin film grown under these conditions has an indistinct orientation. This result is consistent with the SEM images of the diamond thin film prepared at a 2% methane concentration, as shown in Figure 3a.

As the methane concentration increases (3–4%), the preferential orientation effect of the diamond thin film becomes more evident. The proportion of the (111) crystal plane continues to increase, and the full width at half maximum (FWHM) gradually decreases, suggesting that the diamond grain size is increasing and the crystal plane profile is becoming clearer. However, when the methane concentration increases to 5%, the change in the intensity of the (111) diffraction peak is relatively small, and the FWHM increases. This is mainly because the increased methane concentration leads to a higher number of carbon-containing active species in the plasma, which promotes the occurrence of secondary nucleation in the diamond thin film, thereby reducing the crystal quality of the film.

**Figure 6:**
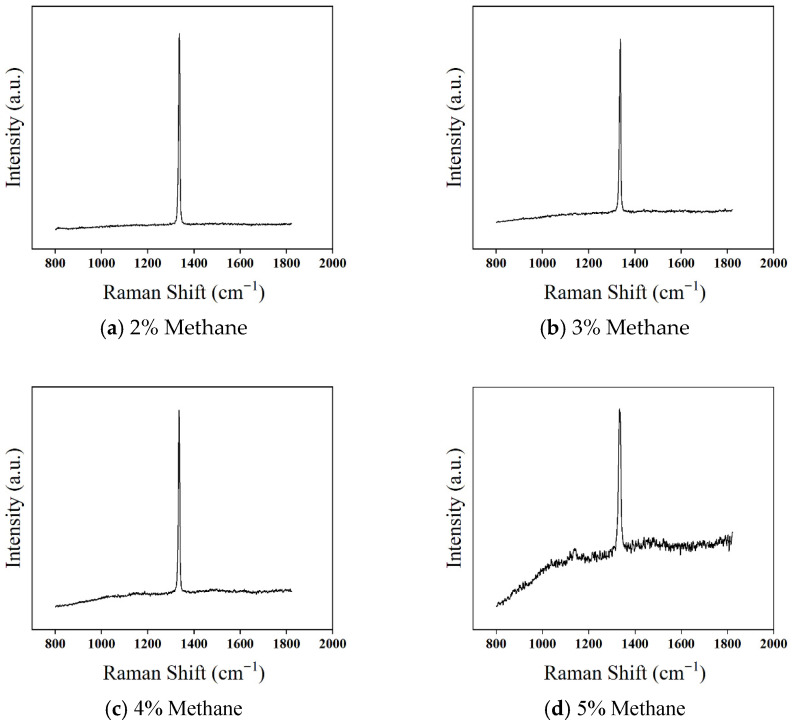
Raman Spectra of Diamond Thin Films Prepared at Different Methane Concentrations.

To further study the impact of methane concentration on the quality of the diamond thin film, Raman spectroscopy was used for characterization and analysis. Previous research has shown that the scattering cross-section of graphite is 50–200 times that of diamond, and Raman spectroscopy is much more sensitive to the graphite phase than to the diamond phase. Even trace amounts of graphite in the diamond thin film can be detected. Figure 6 shows the Raman spectra of diamond thin films prepared under different methane concentrations. It can be seen that for methane concentrations between 2% and 5%, the Raman spectra of the diamond thin films exhibit a distinct diamond first-order Raman peak. However, as the methane concentration increases, the FWHM of the diamond Raman peak gradually broadens, and fluorescence noise (photoluminescence (PL) background) becomes more apparent. This indicates a decrease in diamond crystal quality, worsening crystallinity, and an increase in non-diamond carbon content.

The main reason for this phenomenon is that as the methane concentration increases, the number of carbon-containing active species also increases. While this promotes diamond thin-film deposition, it also leads to the formation of non-diamond carbon phases. Additionally, the proportion of hydrogen atoms in the plasma decreases, reducing the etching rate of non-diamond carbon, which ultimately results in a decline in the crystal quality of the diamond thin film.

### 3.2. Effect of Methane Concentration on Diamond Film Growth

As shown in Figure 6a, when the methane concentration is 2%, the first-order characteristic Raman peak of diamond exhibits high intensity, the PL background remains smooth, and no impurity peaks appear. The (FWHM) is 6.1 cm^−1^, which is very close to the theoretical lower limit of 5 cm^−1^ for heteroepitaxial CVD polycrystalline diamond films. This indicates excellent crystallinity and low impurity content in the diamond film.

As the methane concentration increases to 3%, as shown in Figure 6b, the first-order diamond Raman peak remains strong, but the FWHM increases to 6.5 cm^−1^. Additionally, the PL background starts to rise from the low-frequency end toward the high-frequency region. Studies have shown that the content of non-diamond carbon is positively correlated with the PL background intensity of the diamond film, indicating an increase in non-diamond carbon content in the film.

When the methane concentration further increases to 4%, as shown in Figure 6c, the intensity of the first-order diamond Raman peak does not weaken, but the PL background intensity increases, and the FWHM expands to 7.3 cm^−1^. This phenomenon occurs because, as the methane concentration rises, the hydrogen atom content in the plasma gradually decreases while the concentration of carbon-containing reactive species increases. Consequently, the etching rate of non-diamond carbon by atomic hydrogen falls below its deposition rate, leading to a significant increase in non-diamond carbon content and a decline in crystalline quality.

As depicted in Figure 6d, at a methane concentration of 5%, the Raman spectrum of the diamond film shows substantial changes compared to the previous concentrations. The intensity of the first-order diamond Raman peak decreases significantly, the PL background intensity rises sharply, and the FWHM broadens to 11.6 cm^−1^. This dramatic broadening primarily results from a substantial increase in non-diamond carbon content, reducing the long-range ordered diamond grain domains (i.e., the average defect-free diamond crystal domains) and degrading film crystallinity. Additionally, a Raman peak at 1140 cm^−1^ corresponding to trans-polyacetylene (TPA) and a broad peak at 1500 cm^−1^ indicative of non-diamond carbon appear. These observations confirm that at 5% methane concentration, the diamond film experiences a rapid increase in non-diamond carbon content and a decline in crystalline quality.

From the Raman spectra, it is evident that when the methane concentration increases from 2% to 4%, the overall crystalline quality of the diamond film remains relatively high, despite a slight decrease. However, at 5% methane concentration, the film’s crystallinity deteriorates significantly, and impurity content rises. Furthermore, at extremely low methane concentrations (typically below 1%), the number of carbon-containing reactive species is insufficient, while the atomic hydrogen content in the plasma is excessively high. This imbalance leads to an extremely slow diamond growth rate or even a net etching effect, making it difficult to deposit a continuous and dense diamond film.

In summary, at lower methane concentrations (2–3%), the number of carbon-containing reactive species in the plasma is relatively low, while the hydrogen atom content is high. As a result, the diamond films exhibit small grain sizes with unclear contours and slow deposition rates. However, due to the extensive etching effect of hydrogen atoms, the non-diamond carbon content remains low, yielding excellent crystalline quality. Conversely, at excessively high methane concentrations (5%), the number of carbon-containing reactive species is excessive, leading to rapid film growth. However, the reduced hydrogen atom content facilitates secondary nucleation, significantly degrading film quality. Based on the characterization results, a methane concentration of 4% provides the optimal balance, yielding a continuous, dense diamond film with high crystallinity, low impurity content, well-defined grain contours, and a relatively high growth rate, making it the preferred choice for diamond film fabrication.

### 3.3. Effect of Deposition Temperature on Diamond Film Growth

During the fabrication of diamond films via the microwave plasma chemical vapor deposition (MPCVD) method, deposition temperature fundamentally influences the growth process, playing a decisive role in the grain morphology, crystallographic orientation, and crystalline quality of the film. Studies have shown that the typical growth temperature for CVD diamond films ranges from 600 °C to 1200 °C. When the deposition temperature is too low, the dissociation of gas precursors is insufficient, and the plasma energy is relatively low. This results in inadequate growth and etching driving forces, leading to a decline in film quality. Conversely, when the deposition temperature is excessively high, severe graphitization of the diamond may occur.

In this section, the effect of different deposition temperatures (700 °C, 800 °C, 900 °C, and 950 °C) on diamond film growth is investigated while keeping other process parameters constant. Through the characterization of film quality and morphology, the optimal deposition temperature for high-quality diamond film growth is determined. The specific experimental parameters are shown in Table 3.

Once all process gases are introduced, the increase in deposition temperature is achieved by adjusting the microwave power and chamber pressure. A balance between microwave power and chamber pressure must be maintained; otherwise, excessive reflected power may occur, leading to plasma instability, such as the formation of plasma fireballs. Due to the equipment’s microwave power limitations, it is challenging to reach a deposition temperature of 1000 °C. Therefore, the deposition temperature for sample D is set at 950 °C.

**Figure 7:**
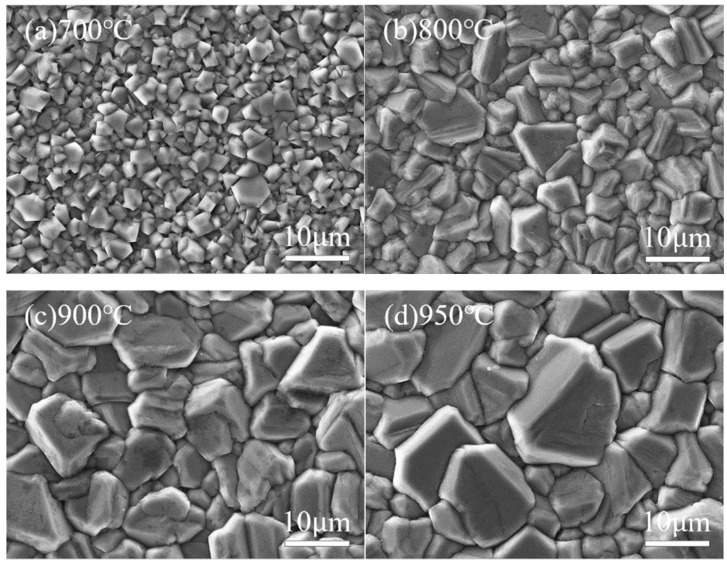
SEM Images of Diamond Films Deposited at Different Temperatures.

**Figure 8:**
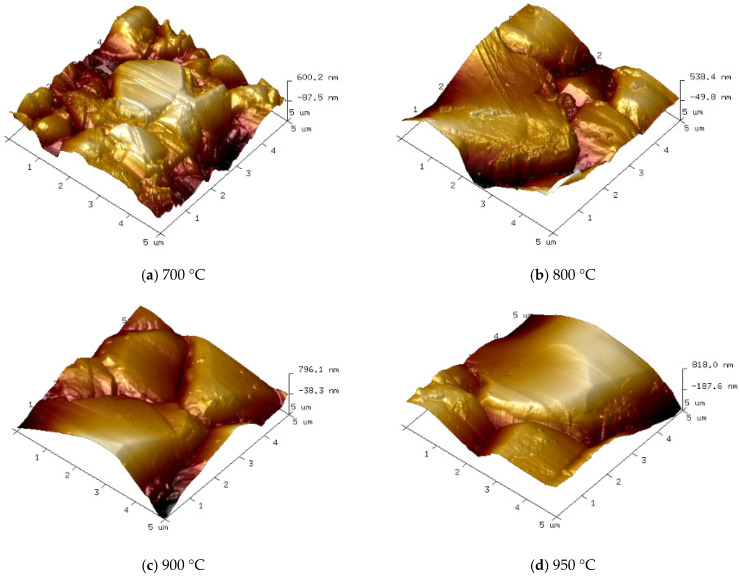
Three-Dimensional Surface Morphology AFM Images of Diamond Films Deposited at Different Temperatures (5 μm × 5 μm).

Figure 7 and Figure 8 show the surface morphology SEM images and corresponding 3D AFM images of diamond films deposited at different temperatures. It is evident that deposition temperature significantly influences the surface morphology of the diamond films.

At a deposition temperature of 700 °C, as shown in Figure 7a and Figure 8a, the diamond film appears continuous and dense, with a surface roughness of Ra = 125 nm. However, the grain size is relatively small (approximately 2 μm on average), and the grain contours are indistinct. This phenomenon is mainly attributed to the low deposition temperature, which results in insufficient gas dissociation and lower plasma energy, leading to a weak driving force for diamond film growth.

As the deposition temperature increases to 800 °C, as shown in Figure 7b and Figure 8b, the grain size of the diamond film increases significantly (average size of 6 μm), and the contours become more distinct. However, grain uniformity decreases slightly, and surface roughness increases to Ra = 147 nm. This is due to the higher deposition temperature enhancing both the driving force for diamond film growth and the etching capability of atomic hydrogen against non-diamond carbon phases.

As shown in Figure 7c and Figure 8c, when the deposition temperature increases to 900 °C, the diamond film exhibits uniform grain size (approximately 10 μm), well-defined contours, and distinct grain boundaries. The surface roughness increases to Ra = 163 nm, indicating that the higher temperature enhances gas dissociation, generating more atomic hydrogen and reactive carbon species, which effectively improves the quality of the diamond film.

However, when the temperature further rises to 950 °C, as seen in Figure 7d and Figure 8d, the grain size continues to increase (average size of approximately 13 μm), with more pronounced grain boundaries and sharper contours. At this stage, the surface roughness reaches Ra = 171 nm. Nevertheless, the preferential orientation growth mechanism of the diamond film restricts the growth of other grains, leading to reduced grain uniformity.

By characterizing the cross-section of the samples using a scanning electron microscope (SEM), the growth rates of diamond films at different deposition temperatures were obtained. The growth rates corresponding to deposition temperatures of 700 °C, 800 °C, 900 °C, and 950 °C were 2.3 μm/h, 3.5 μm/h, 4.6 μm/h, and 5.1 μm/h, respectively. It was observed that within a certain temperature range, the growth rate of the diamond film increased with rising deposition temperature. This is primarily due to the enhanced gas dissociation at higher temperatures, which increases the concentration of atomic hydrogen and carbon-containing active species in the plasma, thereby enhancing the driving force for film growth. However, when the temperature becomes excessively high, the diamond film gradually transitions to a graphitic phase, significantly reducing its crystalline quality.

**Figure 9:**
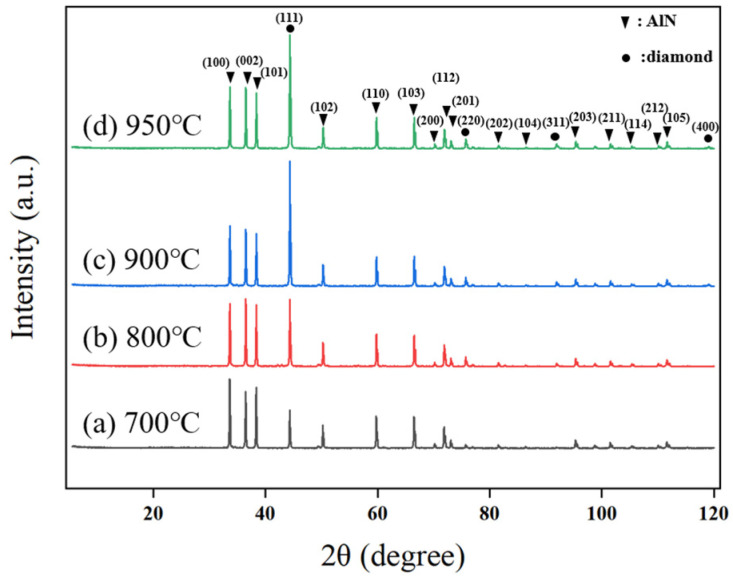
XRD Patterns of Diamond Films Deposited at Different Temperatures.

Similarly, changes in deposition temperature also have a critical impact on the crystallographic orientation of diamond films. As shown in Figure 9, the diamond film predominantly grows along the (111) plane during deposition. At a deposition temperature of 700 °C, only (111) and (220) diffraction peaks appear, with relatively low intensity, indicating weak orientation and unclear grain boundaries. When the temperature increases to 800 °C, the intensity of the (111) diffraction peak increases, and a (311) peak emerges, suggesting a gradual enhancement in preferential orientation and clearer grain contours. At 900 °C, the (400) diffraction peak appears, and the (111) peak intensity significantly increases, indicating a stronger preferential orientation, with the film tending toward a single dominant crystalline orientation. However, when the temperature rises further to 950 °C, the intensity of the (111) peak slightly decreases, primarily due to the formation of non-diamond carbon phases at excessively high temperatures, which degrades the crystalline quality of the film.

**Figure 10:**
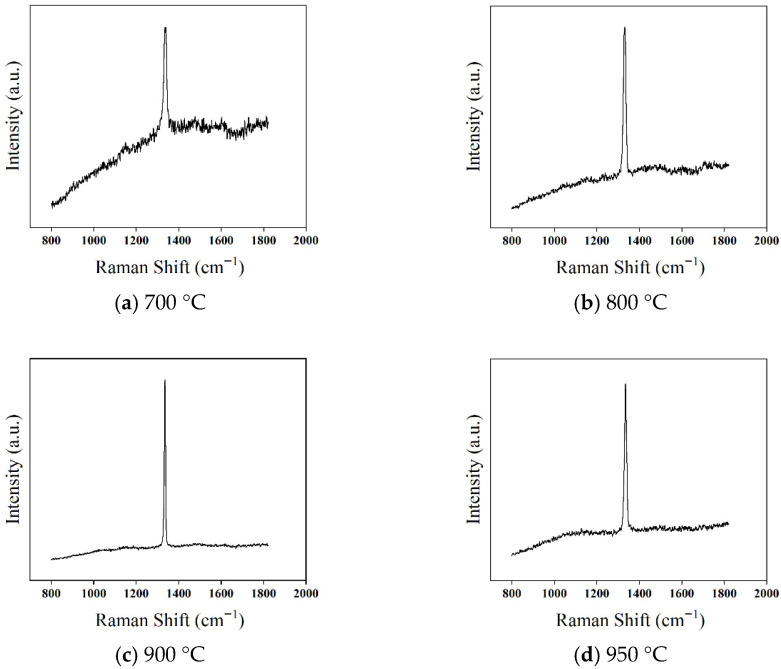
Raman Spectra of Diamond Films Deposited at Different Temperatures.

To further investigate the effect of deposition temperature on the quality of diamond films, Raman spectroscopy was used to characterize the films, as shown in Figure 10. The Raman spectra of diamond films deposited at four different temperatures all exhibit a distinct first-order diamond characteristic peak near 1332.5 cm^−1^.

At a deposition temperature of 700 °C (Figure 10a), the first-order diamond Raman peak is relatively weak, accompanied by a strong (PL) background. The (FWHM) is 13.7 cm^−1^, and a broad Raman peak appears near 1500 cm^−1^, indicating poor crystallinity and a high content of non-diamond carbon phases. This phenomenon is primarily due to the low deposition temperature, which results in insufficient gas dissociation, a limited number of excited hydrogen atoms with lower energy, and weaker etching of non-diamond carbon. Consequently, the deposited diamond film contains more impurities and defects, leading to lower crystal quality.

As the temperature increases to 800 °C (Figure 10b), the PL background is significantly reduced, and the intensity of the first-order diamond Raman peak increases, while the FWHM decreases to 12.4 cm^−1^. However, a weak broad peak at 1500 cm^−1^ remains, indicating that although the higher temperature enhances the etching effect of hydrogen atoms on non-diamond carbon phases, the etching rate is still lower than the deposition rate of the non-diamond phase.

At 900 °C (Figure 10c), the intensity of the first-order diamond Raman peak increases further, the FWHM narrows to 7.3 cm^−1^, and the PL background becomes smoother. This suggests that the enhanced hydrogen etching effectively suppresses the formation of non-diamond carbon, improving the film quality.

However, when the deposition temperature rises to 950 °C (Figure 10d), the first-order diamond Raman peak intensity decreases, the PL background reappears, and the FWHM increases to 9.8 cm^−1^. This is mainly attributed to the desorption of hydrogen atoms from the film surface at higher temperatures. As a result, free sp^2^ carbon groups interact with the dangling bonds of surface carbon atoms, leading to a gradual transformation from sp^3^ diamond bonds to sp^2^ bonds, increasing the non-diamond carbon content and degrading the film quality.

In summary, at lower deposition temperatures (700–800 °C), the degree of gas dissociation is insufficient, resulting in a lower concentration and energy of carbon-containing active species and hydrogen atoms in the plasma. This leads to a slow deposition rate, small and indistinct grain morphology, and poor crystalline quality of the diamond films. At higher deposition temperatures (950 °C), although the increased plasma energy density enhances the deposition rate, the grain uniformity deteriorates. Additionally, the partial desorption of hydrogen atoms from the film surface increases the non-diamond carbon content, ultimately reducing the crystalline quality. Considering both the growth rate and crystal quality of the diamond films, a deposition temperature of 900 °C is optimal for achieving well-defined grain boundaries, uniform grain size, clear morphology, a relatively high growth rate, and superior crystal quality.

### 3.4. Effect of Oxygen Flow Rate on Diamond Film Growth

During the MPCVD growth of diamond films, the introduction of oxygen [23] can significantly influence the surface morphology and crystallographic orientation of the films. Studies have shown that adding an appropriate amount of oxygen to the CH_4_-H_2_ gas mixture can effectively suppress the deposition of non-diamond carbon phases, thereby improving the crystalline quality of the diamond film.

In this section, we investigate the effect of different oxygen flow rates (0.5 sccm, 1 sccm, and 1.5 sccm) on the growth quality, surface morphology, and deposition rate of diamond films while keeping other process parameters constant. The specific experimental parameters are listed in Table 4, with sample “a” serving as the control group without oxygen addition.

**Figure 11:**
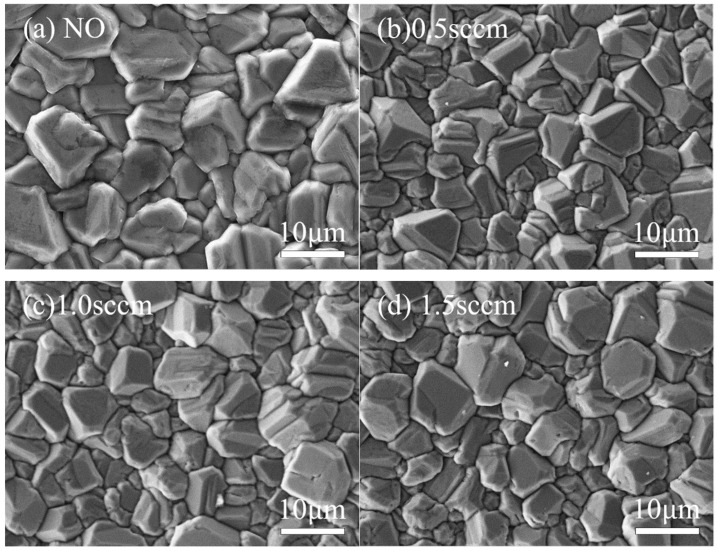
SEM Images of Diamond Films Grown at Different Oxygen Flow Rates.

**Figure 12:**
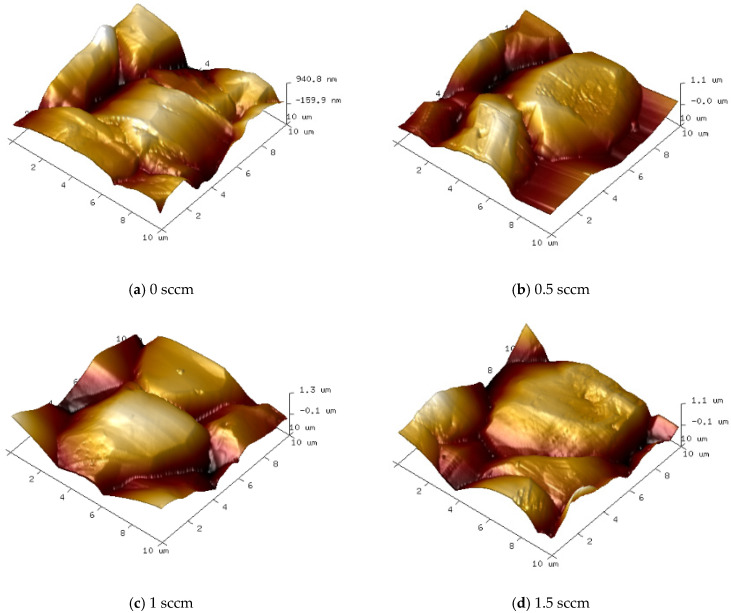
Three-Dimensional AFM Images of Diamond Film Surfaces Grown at Different Oxygen Flow Rates (10 μm × 10 μm).

Under different oxygen flow rate conditions, the surface morphology and grain aggregation of diamond films exhibit distinct characteristics. Figure 11 and Figure 12 show SEM images of the surface morphology and corresponding AFM 3D topography images of diamond films grown at varying oxygen flow rates.

As shown in Figure 11b and Figure 12b, when the oxygen flow rate is 0.5 sccm, the diamond film remains continuous and dense, with well-defined grain boundaries and a clear outline. The surface roughness is measured at Ra = 152 nm. However, compared to the sample without oxygen, the average grain size decreases to approximately 8 μm, and the grain morphology gradually shifts from a pyramidal shape to a more cubic form. At an oxygen flow rate of 1.0 sccm, as shown in Figure 11c and Figure 12c, the grain size remains similar to that of the 0.5 sccm sample, and the film maintains good density. The surface roughness decreases to Ra = 141 nm. However, a greater proportion of pyramidal grains transform into cubic ones, and some grain surfaces display scratch-like features caused by oxygen etching. When the oxygen flow rate increases to 1.5 sccm, as seen in Figure 11d and Figure 12d, the proportion of cubic-shaped diamond grains increases, but their size uniformity decreases. At this point, the surface roughness slightly rises to Ra = 146 nm, and many smaller grains exhibit uneven surfaces with blurred contours. This indicates that as the oxygen flow rate increases, the etching effect of oxygen atoms on the diamond phase is enhanced.

By using a high-precision thickness gauge to measure the sample thickness, the diamond film growth rates at different oxygen flow rates were obtained. When the oxygen flow rate was 0.5 sccm, the growth rate of the diamond film slightly increased from 4.1 μm/h (without oxygen) to 4.3 μm/h. The primary reason is that the addition of oxygen to the CH_4_/H_2_ plasma environment leads to the generation of OH radicals, which effectively promote the ionization and decomposition of methane, thereby increasing the concentration of CH_3_ active species necessary for diamond film growth. Additionally, OH radicals facilitate the desorption of hydrogen atoms from the diamond film surface, generating more dangling carbon bonds, which further enhances the effective growth rate of the diamond film:

(3) $$\begin{array}{c} {CH}_{4}+O\to {CH}_{3}+OH \end{array}$$

(4) $$\begin{array}{c} {CH}_{4}+OH\to {CH}_{3}+H_{2}O \end{array}$$

(5) $$\begin{array}{c} C_{d}-H+OH\to C_{d}^{*}+H_{2}O \end{array}$$

When the oxygen flow rate increased to 1.0 sccm, the diamond film growth rate decreased to 3.8 μm/h. This phenomenon is primarily due to the enhanced oxidation reactions of CH_3_ by oxygen and atomic oxygen, as shown in Equations (6) and (7). After oxidation, CH_3_ forms stable compounds such as HCHO, CO, and CO_2_, leading to a reduction in the concentration of the CH_3_ precursor required for diamond growth. Additionally, the increased oxygen flow raises the atomic oxygen concentration in the plasma, intensifying the etching effect on the diamond phase. The combined impact of these two factors hinders diamond film deposition.

When the oxygen flow rate further increased to 1.5 sccm, the diamond film growth rate dropped to 3.4 μm/h, indicating that the increased oxygen content significantly enhanced the etching effect on the diamond phase. In extreme cases, when the etching rate of H and O reaches or exceeds the deposition rate, the diamond film growth may be completely suppressed.

(6) $$\begin{array}{c} {\mathrm{CH}}_{3}+O\to HCHO+H\; \end{array}$$

(7) $${\mathrm{CH}}_{3}+O/O_{2}\to \mathrm{CO}/{\mathrm{CO}}_{2}$$

**Figure 13:**
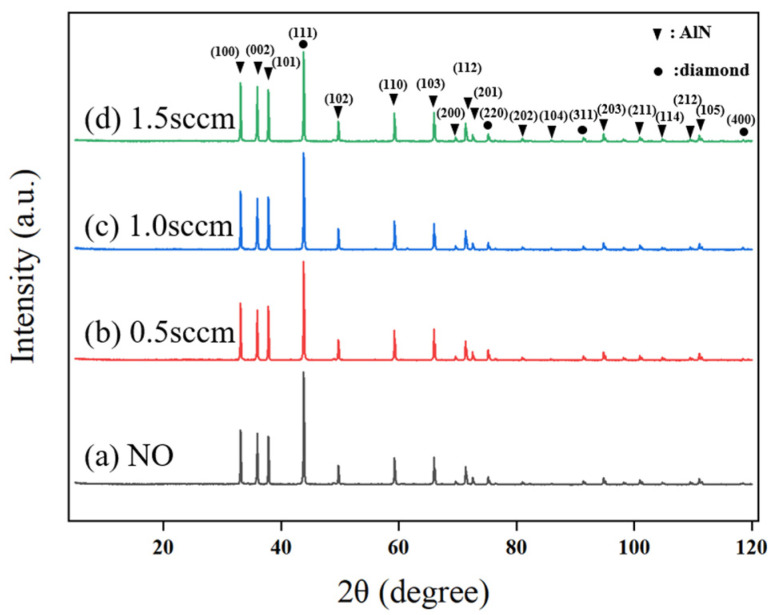
XRD Patterns of Diamond Films Prepared at Different Deposition Temperatures.

From SEM observations, the introduction of oxygen into the growth gas mixture significantly alters the surface morphology of the diamond film. To further understand the impact of oxygen on crystal orientation, XRD analysis was performed on the diamond films, with results shown in Figure 13. It is evident that after oxygen is introduced, the diamond film continues to exhibit a preferential growth orientation along the (111) plane. However, as the oxygen flow rate increases, the intensity and proportion of the (111) diffraction peak gradually decrease, accompanied by a slight increase in the (FWHM). This indicates that the presence of oxygen reduces both the grain size and the preferential orientation of the diamond film.

At an oxygen flow rate of 0.5 sccm, although the intensity of the (111) diffraction peak decreases, the intensity of the (220) diffraction peak slightly increases. However, when the oxygen flow rate is further increased to 1.0 sccm and 1.5 sccm, the (220) diffraction peak intensity decreases again. This suggests that a small amount of oxygen shifts the preferential orientation of the diamond film from (111) to (220), but with a further increase in oxygen flow, the (111) orientation regains dominance.

**Figure 14:**
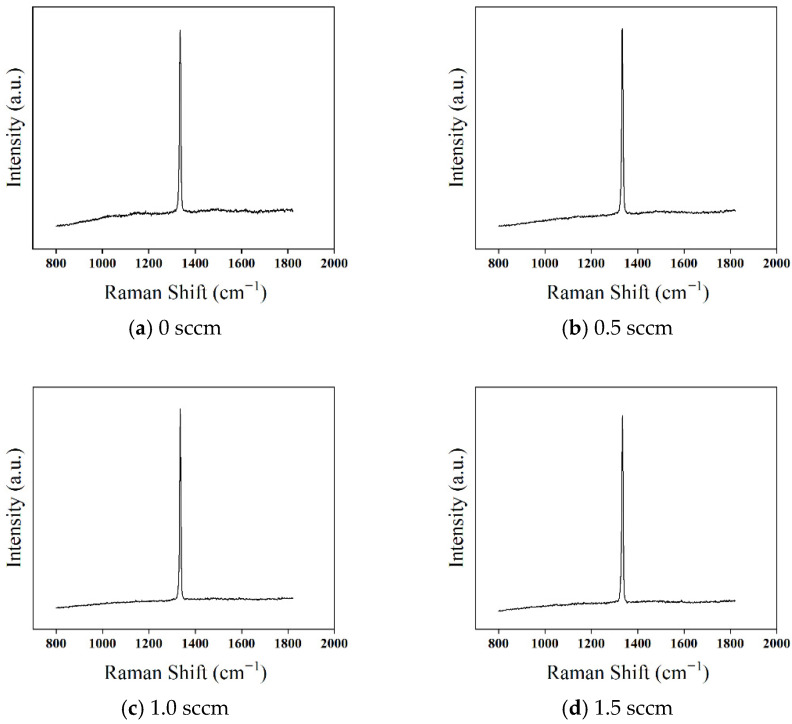
Raman Spectra of Diamond Films Deposited at Different Oxygen Flow Rates.

To further investigate the effect of oxygen addition on the quality of diamond films, Raman spectroscopy was used for characterization, with results presented in Figure 14. The Raman spectra for all three oxygen flow conditions exhibit a strong first-order diamond Raman peak at 1332.5 cm^−1^, without any additional peaks corresponding to non-diamond phases.

When the oxygen flow rate is 0.5 sccm, the (PL) background in the Raman spectrum becomes smoother compared to the sample without oxygen, and the FWHM of the diamond peak decreases from 7.3 cm^−1^ to 6.7 cm^−1^. This indicates that oxygen suppresses the deposition of non-diamond carbon phases. The reason for this phenomenon is that the deposition process of non-diamond carbon is similar to that of diamond, primarily occurring through the reaction of C_2_H_2_ molecules with surface carbon atoms via hydrogen abstraction, forming dangling bonds.

(8) $$\begin{array}{c} C_{g}\cdot +C_{2}H_{2}\to C_{g}C_{2}H_{2} \end{array}$$

(9) $$\begin{array}{c} C_{g}\cdot +C_{2}H_{2}\to C_{g}C_{2}H+H \end{array}$$

The addition of oxygen promotes the dissociation of CH_4_, leading to the generation of more CH_3_ radicals, which in turn reduces the concentration of C_2_H_2_. This results in a lower deposition rate of non-diamond carbon phases. Additionally, some of the byproducts of the hydrogen etching reaction on non-diamond carbon also include C_2_H_2_. As the concentration of C_2_H_2_ decreases, the etching reaction intensifies, further suppressing the formation of non-diamond carbon. Moreover, reactive species such as O_2_, O, and OH present in the plasma contribute to the direct etching of non-diamond carbon. These combined effects lead to a reduction in impurity content, thereby improving the crystal quality of the diamond film.

When the oxygen flow rate is further increased to 1.0–1.5 sccm, the (PL) background in the Raman spectrum continues to weaken, while the FWHM values remain relatively stable at 6.5 cm^−1^ and 6.3 cm^−1^, respectively. This indicates that the suppression of non-diamond carbon deposition becomes more pronounced with increasing oxygen concentration, further enhancing the crystal quality of the diamond film.

In summary, introducing an appropriate amount of oxygen into the CH_4_/H_2_ gas mixture significantly alters the surface morphology of diamond films. A lower oxygen flow rate (0.5 sccm) slightly increases the deposition rate of the diamond film while suppressing the formation of non-diamond carbon, leading to improved film quality. However, the preferential growth of the (111) crystal plane diminishes slightly, and the grain size decreases. At higher oxygen flow rates (1.0–1.5 sccm), the suppression of non-diamond carbon becomes more pronounced, further enhancing the crystalline quality of the film. However, the etching effect of O_2_, O, and OH on the diamond phase reduces the film deposition rate.

### 3.5. LED Junction Temperature and Thermal Resistance Testing on Different Packaging Substrates

**Figure 15:**
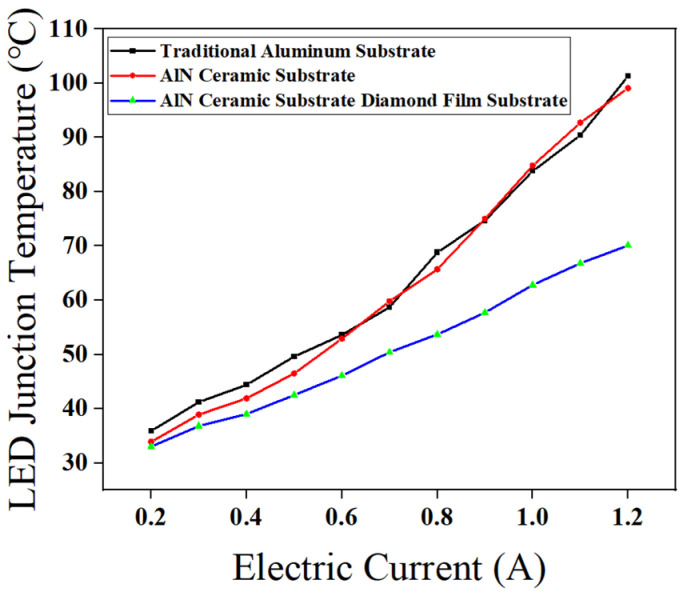
Line graph of LED junction temperature under different currents.

The thermal conductivity of polycrystalline diamond [36,37,38] is restricted by grain boundary scattering. Therefore, by carefully controlling the growth parameters (CH_4_ concentration, deposition temperature and oxygen flow rate), a high-quality diamond layer with continuous and compact diamond film, good grain uniformity and low impurity content was prepared, and the preferred orientation of 111 crystal proved the reliability of this growth process. In order to evaluate the heat dissipation [39,40,41,42,43] performance of diamond film substrate with AlN ceramic substrate, the traditional aluminum substrate and AlN ceramic substrate were compared with the diamond film substrate with AlN ceramic substrate prepared by the optimal process, and the LED junction temperature changes of the three substrates were measured under different current drives when the temperature stabilized. The results are shown in Figure 15.

The results indicate that as the driving current increases, the junction temperature of LEDs on all three substrates exhibits an upward trend. However, the rate of temperature rise for LEDs packaged on traditional aluminum and AlN ceramic substrates is significantly higher than that of LEDs packaged on the AlN ceramic substrate with a diamond film.

**Figure 16:**
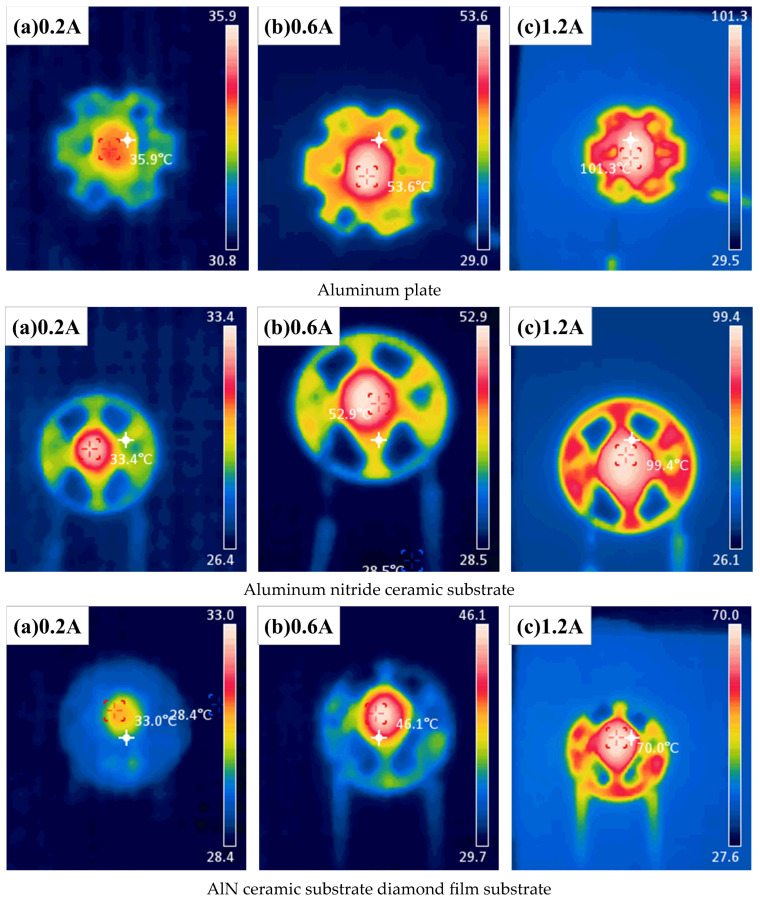
Infrared Imaging of LED Junction Temperature under Substrate Packaging.

Figure 16: Infrared Imaging of LED Junction Temperatures with Different Substrates, when the initial current is set to 0.2 A, the LED packaged with an AlN ceramic substrate with a diamond thin film exhibits the lowest junction temperature at 33 °C. Although aluminum has a higher thermal conductivity than AlN ceramics, the LED using a conventional aluminum substrate records the highest junction temperature among the three at 35.9 °C. This phenomenon is primarily attributed to the low thermal conductivity insulating resin layer coated on the aluminum substrate, which leads to heat accumulation. The infrared imaging clearly reveals that most of the heat in the conventional aluminum substrate is trapped within the insulating resin layer, creating a noticeable temperature difference between the exposed aluminum and the resin-coated regions.

As the current increases to 0.6 A, the rising power of the LED chip causes an overall increase in the packaging temperature. The AlN ceramic substrate LED shows the largest temperature rise of 19.5 °C, followed closely by the conventional aluminum substrate with a rise of 17.7 °C. Meanwhile, the AlN ceramic substrate with a diamond thin film demonstrates the smallest increase at 13.1 °C.

When the current is further increased to 1.2 A, the junction temperatures of LEDs packaged with the conventional aluminum substrate, AlN ceramic substrate, and AlN ceramic substrate with a diamond thin film reach 101.3 °C, 99.4 °C, and 70 °C, respectively. Compared to the conventional aluminum and AlN ceramic substrates, the AlN ceramic substrate with a diamond thin film reduces the LED junction temperature by 30.9% and 29.6%, respectively.

Furthermore, the temperature rise compared to the initial 0.2 A condition is 65.4 °C, 66 °C, and 36 °C for the conventional aluminum substrate, AlN ceramic substrate, and AlN ceramic substrate with a diamond thin film, respectively. These results highlight the superior thermal dissipation [44] capability of the AlN [45,46] ceramic substrate with a diamond thin film, effectively reducing the junction temperature of high-power LEDs. Notably, this advantage becomes even more pronounced as the LED power increases.

As the temperature rises, it is evident from the infrared imaging that there is a significant temperature difference between the metallized region and the main body of both the AlN ceramic substrate and the AlN ceramic substrate diamond [47] film. Studies have shown that when an LED is powered, the generated heat propagates both vertically and horizontally through the substrate, creating a one-dimensional thermal resistance in the vertical direction and a spreading thermal resistance in the horizontal direction. Together, these components contribute to the overall thermal resistance of the packaging substrate. Since both AlN ceramics and diamond primarily conduct heat through phonon transport, they exhibit low one-dimensional thermal resistance, allowing heat generated by the LED to be rapidly transferred in the vertical direction. Consequently, the metal layer on the substrate’s surface does not accumulate sufficient heat to increase its temperature, leading to noticeable temperature differences.

In contrast, aluminum substrates consist of multiple layers (insulating resin—conductive layer—insulating layer—metal layer), where interfacial thermal resistance significantly hinders heat transfer. The restricted vertical heat dissipation results in heat accumulation along the horizontal plane, which aligns with the observation in Figure 16, where the insulating resin region of the aluminum substrate exhibits higher temperatures. Thermal resistance [48] is defined as the ratio of the temperature difference between two reference points along a heat conduction path to the heat conversion power of the heat source (i.e., the rate of heat transfer between the two points). It reflects the material’s ability to resist heat flow:

(10) $$\begin{array}{c} R_{th}=\frac{\Delta T}{q} \end{array}$$

In the equation:

$R_{th}$ represents the thermal resistance between two reference points (°C/W).
Δ*T* is the temperature difference between the two reference points (°C).
*q* is the heat transfer rate between the two reference points (W).

For the three different LED packaging substrates in this study, thermal resistance [31] serves as a key parameter reflecting the ability of the LED device to impede heat transfer. The thermal resistance value is defined as the ratio of the temperature difference between the LED junction and the external environment to the heat transfer rate. The LED junction temperature is obtained from the highest temperature recorded by the infrared thermometer. However, the exact heat transfer rate is difficult to determine, so 80% of the LED device’s power is typically used as a reference.

To better evaluate the heat dissipation performance of the substrates in LED devices, this experiment focuses on calculating the thermal resistance from the LED chip to the substrate. The bottom surface temperature of the substrate is measured using a thermocouple, and the obtained thermal resistance value represents the combined thermal resistance between the LED chip and the substrate. The results are shown in Table 5.

Since the thermal resistance of the heat dissipation substrate is primarily determined by the material itself and is largely unaffected by the heat generated by the LED device, the comprehensive thermal resistance of the aluminum substrate is higher than that of the AlN ceramic substrate. This is despite aluminum having a higher thermal conductivity than AlN ceramics. The key reason is that the AlN ceramic substrate uses a high thermal conductivity metal as its conductive layer, and its thickness is significantly smaller than the insulating layer of the aluminum substrate.

When the LED device operates at a power of 3 W, the AlN ceramic substrate with a diamond thin film exhibits the lowest comprehensive thermal resistance. Compared to the traditional aluminum substrate and the AlN ceramic substrate, its thermal resistance is reduced by 10.2 K/W and 3.9 K/W, respectively.

## 4. Conclusions

In this paper, diamond films were prepared on AlN ceramic substrates by MPCVD method. The effects of process parameters on the growth of the films were systematically studied, and the heat dissipation performance of the metallized packaged LED devices was tested. The main conclusions are as follows:

1. When the concentration of methane is 4%, the deposition rate of diamond film is faster, the crystal quality is good, and the surface is dense and uniform;
2. When the deposition temperature is 900 °C, diamond films with uniform grains, clear outline, high crystallization quality and excellent growth rate can be obtained;
3. Low flow rate of oxygen (0.5 sccm) is helpful to improve the deposition rate and inhibit the formation of non-diamond phase, while high flow rate (1.0–1.5 sccm) leads to a significant decrease in the deposition rate due to etching;
4. Compared with aluminum substrate and AlN ceramic substrate, AlN/diamond composite substrate shows better heat dissipation performance. Under the working current of 1.2 A, the junction temperature of LED decreases by 30.9% and 29.4%. At the power of 3 W, the thermal resistance decreased by 10.2 K/W and 3.9 K/W, respectively, indicating that the diamond film significantly enhanced the thermal conductivity of AlN ceramics.

## Figures and Tables

**Table 1:** Deposition parameters of diamond thin films.

Substrate Temperature/°C	Methane Concentration/%	O_2_/sccm	Microwave Power/W	Sedimentary Pressure/Torr
900	4%	0.5	4600	155

**Table 2:** Growth Parameters of Diamond Thin Films at Different Methane Concentrations.

Sample	Microwave Power (W)	Deposition Temperature (°C)	Chamber Pressure (Torr)	Hydrogen Flow Rate (sccm)	Deposition Time (h)	Methane Concentration (%)
a	4600	900	155	500	4	2
b						3
c						4
d						5

**Table 3:** Growth Parameters of Diamond Films at Different Deposition Temperatures.

Sample	Hydrogen Flow Rate (sccm)	Methane Concentration (%)	Growth Duration (h)	Deposition Temperature (°C)
a	900	4	4	700
b				800
c				900
d				950

**Table 4:** Growth Parameters of Diamond Films Under Different Oxygen Flow Rates.

Sample	Deposition Temperature (℃)	Hydrogen Flow Rate (sccm)	Methane Concentration (%)	Deposition Duration (h)	Oxygen Flow Rate (sccm)
a	900	500	4	4	0
b					0.5
c					1.0
d					1.5

**Table 5:** Thermal Resistance of Different Substrates.

Substrate Type	Power/W	Current/A	Voltage/V	LED Junction Temperature/℃	Total Thermal Resistance (K/W)
Traditional Aluminum Substrate	3	1	3	83.8	14.1
AlN Ceramic Substrate				84.5	7.8
AlN Ceramic Substrate with Diamond Thin Film				62.4	3.9

## Data Availability

The original contributions presented in this study are included in the article. Further inquiries can be directed to the corresponding author.
